# Association Between Exercise Adherence and Long‐Term Quality of Life in Ischemic Stroke Survivors: A 2‐Year Prospective Cohort Study

**DOI:** 10.1002/nop2.70965

**Published:** 2026-10-30

**Authors:** Jin Ziyuan, Zhang Hong, Liu Zhili, Wu Yanchun, Lin Hongchun

**Affiliations:** ^1^ Operating Room & Anesthesiology Department The First Affiliated Hospital of Shantou University Medical College Shantou China; ^2^ Nursing Department The First Affiliated Hospital of Shantou University Medical College Shantou China; ^3^ Nursing Department Shantou University Mental Health Center Shantou China; ^4^ Institute of Nursing Research The First Affiliated Hospital of Shantou University Medical College Shantou China; ^5^ Department of Urology The First Affiliated Hospital of Shantou University Medical College Shantou China

**Keywords:** exercise adherence, nursing, quality of life, rehabilitation, stroke

## Abstract

**Aims:**

This study aimed to investigate factors associated with quality of life within 2 years post‐discharge, with a particular focus on exercise adherence.

**Design:**

A prospective observational cohort study.

**Methods:**

Participants were enrolled during hospitalisation and followed up at discharge and at 1, 3, 6, 12 and 24 months post‐discharge via questionnaires. Linear mixed‐effects models were used to identify factors associated with quality of life.

**Data Sources:**

Data were collected from ischemic stroke patients (discharged from The First Affiliated Hospital of Shantou University Medical College, January to August 2021) using structured questionnaires, medical records and validated scales.

**Results:**

Of 355 enrolled stroke survivors, 242 (68.2%) completed the two‐year follow‐up. Functional disability, exercise adherence, family history of stroke and prior stroke were independently associated with long‐term quality of life.

**Conclusion:**

Greater exercise adherence is independently associated with better quality of life on average over the 24‐month follow‐up period among stroke survivors.

**Implications for the Profession and/or Patient Care:**

Nurses and rehabilitation guidelines should prioritise exercise adherence support during long‐term follow‐up to sustain post‐stroke quality of life.

**Abbreviations**
ADL: activity of daily living
EAQ: Exercise Adherence Questionnaire
ICC: intraclass correlation coefficient
LMM: Linear mixed‐effects model
MAR: missing at random
MCAR: missing completely at random
MICE: multiple imputation by chained equations
mRS: modified Rankin Scale
NIHSS: National Institute of Health stroke scale
QoL: quality of life
REML: restricted maximum likelihood
SS‐QoL: Stroke‐specific quality of life scale

## 1 Introduction

Stroke is a leading cause of death and disability worldwide (Youkee et al. 2024). Up to 50% of stroke survivors experience residual limb dysfunction (Lee et al. 2022) and psychological impairment (Gurková et al. 2023; Waller et al. 2023), leading to moderate‐to‐poor quality of life (QoL) (Bello et al. 2021; Segerdahl et al. 2023; Waller et al. 2023; Wong et al. 2021). QoL is defined as individuals' perception of their position in life in the context of the culture and value systems in which they live and in relation to their goals, expectations, standards and concerns (‘The World Health Organization Quality of Life assessment (WHOQOL): position paper from the World Health Organization’ WHOQOL 1995). Changes in QoL have been associated with long‐term survival (Hu et al. 2023). Identifying the key factors influencing QoL is a critical first step to improving outcomes and may enable healthcare workers, particularly nurses, to develop more accurate and effective rehabilitation plans.

While numerous studies have investigated factors influencing QoL in stroke patients (Ayasrah et al. 2024; Silva et al. 2024), the specific role of exercise adherence remains underexplored. Self‐Determination Theory (Flannery 2017) posits that sustained health behaviours emerge when the needs for autonomy, competence and relatedness are fulfilled, explaining why patients initiate and internalise exercise. However, motivation alone does not guarantee long‐term adherence. Habit formation theory (Gardner 2015) complements this by proposing that behavioural automaticity develops through repeated, context‐consistent practice, clarifying how exercise becomes ingrained over time. Despite this theoretical relevance, the majority of existing cohort studies have been limited to follow‐up periods of ≤ 12 months and have relied on simplistic, unidimensional adherence measures (Barbosa et al. 2022; Wang et al. 2024), thereby likely missing the cumulative, habit‐driven associations with QoL. In contrast, the present study extends follow‐up to 2 years (six time points) and uses a stroke‐specific functional exercise adherence scale that captures both motivational and habitual dimensions. Unlike a previous two‐year follow‐up study that assessed QoL using a generic instrument (SF‐12) and included only exercise frequency and duration as the primary exercise variables (Hou et al. 2017), our study assessed QoL with the Stroke‐Specific Quality of Life Scale (SS‐QoL) and exercise adherence with a comprehensive functional exercise adherence scale and applied a linear mixed‐effects model that included all enrolled participants. To the best of our knowledge, this is among the first longitudinal studies to show that exercise adherence is independently associated with better QoL on average over the 24‐month post‐discharge period. These theory‐informed results highlight the need for rehabilitation programs that not only ignite initial motivation but also foster exercise automaticity, thereby optimizing long‐term recovery outcomes.

## 2 Background

Numerous factors affecting QoL in stroke survivors have been identified, such as age (Segerdahl et al. 2023; Youkee et al. 2024), marital status (Ayasrah et al. 2024; Kim et al. 2023), education (Wijesiri et al. 2023), comorbidity (Diegoli et al. 2023; Rosa et al. 2023), functional independence (Braga et al. 2024), socioeconomic status (Sun et al. 2023). Among these, the degree of physical disability has been consistently associated with QoL (Barbosa et al. 2022; Braga et al. 2024; Diegoli et al. 2023; Gurková et al. 2023; Kim et al. 2023). This association may be partly explained by its relationship with self‐efficacy (Dharma et al. 2023), coping style (van Mierlo et al. 2018) and exercise adherence.

Exercise adherence is defined as ‘a person's adherence with a prescribed or self‐prescribed fitness program’ (Gelineau et al. 2022). Existing evidence indicates that exercise adherence in stroke survivors is shaped by a multidimensional network of determinants spanning psychological, social and environmental domains. Psychological factors including subjective norms, attitudes and perceived behavioural control predict post‐stroke exercise intention, with self‐efficacy representing the most consistent and direct predictor of exercise adherence in community‐dwelling stroke survivors (Lin et al. 2022; Zhou et al. 2023). Rehabilitation expectations and subjective well‐being may also be positively associated with adherence, whereas depressive symptoms and physical impairments are negatively associated with it (Liu et al. 2025; Xing et al. 2025; Zhang et al. 2023). In addition, family‐related factors—particularly caregiver capacity and emotional support—play a crucial role in facilitating home‐based exercise adherence among older stroke patients (Zhang et al. 2023). At the rehabilitation environment level, individually tailored exercise prescriptions, ongoing monitoring and feedback mechanisms and supportive organizational policies have been identified as key enablers of sustained adherence (Liu et al. 2025; Vadas et al. 2022; Zhang et al. 2023). Given the pivotal role of exercise adherence in post‐stroke recovery, a growing body of research has sought to identify its determinants. However, few studies have directly examined the prospective association between exercise adherence and long‐term QoL. To address this research gap, we aimed to explore factors, especially exercise adherence, that are associated with QoL in stroke survivors at discharge and 1, 3, 6, 12 and 24 months post‐discharge. We hypothesized that exercise adherence would be independently associated with long‐term QoL among stroke survivors, after adjustment for demographic factors and functional disability.

## 3 Methods

### 3.1 Study Design

This prospective observational study was conducted at The First Affiliated Hospital of Shantou University Medical College, Guangdong, China. Reporting guideline: STROBE checklist (Supporting Information: STROBE checklist).

### 3.2 Participants and Setting

Patients with ischemic stroke who were discharged from The First Affiliated Hospital of Shantou University Medical College from January 2021 to August 2021 were enrolled. Inclusion criteria were as follows: (a) diagnosis of ischemic stroke confirmed by computed tomography or magnetic resonance imaging of the head, according to the diagnostic criteria of the Guidelines for the Prevention and Treatment of Cerebrovascular Diseases in China (Mingli 2006); (b) age ≥ 18 years; (c) ability to cooperate in completing the questionnaire at discharge and during the follow‐up period; (d) possession of a mobile phone to enable completion of follow‐up; and (e) voluntary participation in the study. Exclusion criteria were as follows: (a) discharge due to critical illness or at the family's request; and (b) transfer to a rehabilitation hospital at discharge.

Withdrawal criteria were as follows: (a) inability to contact the participant after two phone calls per day for three consecutive days; (b) stroke recurrence or death; (c) refusal to continue after the first follow‐up; (d) admission to a rehabilitation hospital after discharge; or (e) hospitalisation for other serious illnesses after the first follow‐up.

### 3.3 Sample Size and Precision

The present study used data from an existing longitudinal cohort of ischemic stroke survivors. Accordingly, the sample size was determined by the number of eligible participants with available follow‐up data rather than by a formal a priori sample size calculation for a linear mixed‐effects model. Given that power calculations for linear mixed‐effects models incorporating repeated measures and natural spline terms are statistically complex and highly dependent on assumptions regarding variance components and within‐person correlation, we prioritized the precision of the estimated associations, as reflected by 95% confidence intervals, over conventional power thresholds. The primary association between exercise adherence and QoL was estimated with reasonable precision (*β* = 0.82, 95% CI: 0.69–0.95) and the confidence interval excluded the null value.

### 3.4 Measurements

Sociodemographic data of participants, including age, gender, education, smoking, alcohol drinking, marital status, employment, primary caregiver, family economic status and family history of stroke, were collected using structured questionnaires. Clinical data, including prior stroke and National Institute of Health Stroke Scale (NIHSS) score, were extracted from medical records. Additional variables, including Activities of Daily Living (ADL) score, exercise adherence, modified Rankin Scale (mRS) grade and QoL, were assessed using the scales described below.

### 3.5 Barthel Index Scale

The Barthel index scale was used to evaluate ADL (Mahoney and Barthel 1965). This scale comprises ten items: feeding, bathing, grooming, dressing, bowel control, bladder control, toilet use, transfers (bed to chair and back), mobility (walking on level surfaces) and stair climbing. Each item is scored according to the level of assistance required, ranging from total dependence to complete independence. The total score ranges from 0 to 100, with higher scores indicating greater functional independence and can be categorized into the following levels of dependency: 0–20 (totally dependent), 25–45 (severely dependent), 50–70 (moderately dependent), 75–95 (slightly dependent) and 100 (independent).

### 3.6 Exercise Adherence Questionnaire

The Exercise Adherence Questionnaire (EAQ), developed by Beilei et al. (2013), was used to assess exercise adherence. This scale is primarily designed for the follow‐up evaluation of functional exercise adherence in stroke survivors after discharge and has demonstrated good reliability and validity. Cronbach's α of the total scale was 0.923 and the intraclass correlation coefficient (ICC) reflecting test–retest reliability varied between 0.788 and 0.850 (Jinwen et al. 2024). The scale contains 14 items, each scored on a 4‐point scale: 1 (not at all), 2 (occasionally), 3 (basically) and 4 (completely). Total scores range from 14 to 56, with higher scores indicating better adherence.

### 3.7 Stroke‐Specific Quality of Life Scale

The SS‐QoL was used to assess patient's QoL (Williams et al. 1999). This instrument was specifically designed for stroke patients and has been translated and validated in multiple languages (Choksi et al. 2025; Pedersen et al. 2018; Yilong et al. 2003). The scale comprises 49 items, each scored on a 5‐point scale, yielding a total score ranging from 49 to 245, with higher scores indicating better QoL. The Chinese version of the SS‐QoL was translated and validated in Chinese stroke survivors by Yilong et al. (2003) and has since been used in numerous subsequent studies (Lu et al. 2024; Xiaoxuan et al. 2024; Wang et al. 2026; Ziyuan et al. 2023).

### 3.8 Modified Rankin Scale

The mRS (van Swieten et al. 1988) was used to assess the degree of disability. This scale provides a simple, validated grading of functional outcomes after stroke. Scores range from 0 to 5, with 0 indicating no symptoms, 1 no significant disability, 2 slight disability, 3 moderate disability, 4 moderately severe disability and 5 severe disability.

### 3.9 Data Collection

Two trained data collectors gathered all study data from participants. Follow‐up data on exercise adherence, QoL and mRS grade were collected at 1, 3, 6, 12 and 24 months post‐discharge.

To ensure data quality, the following measures were implemented: (a) both data collectors underwent standardized training and used consistent wording and explanations during questionnaire administration; (b) to enhance follow‐up adherence and minimise loss to follow‐up, participants were reinformed about the follow‐up procedures and study duration after completing the baseline questionnaire and their contact information was reconfirmed; (c) the same data collection method was used at each time point to reduce bias arising from procedural variations; (d) all data were independently double‐entered and cross‐checked by two researchers using EpiData version 3.1 to minimise data entry errors.

### 3.10 Data Analysis

Statistical analyses were performed using R version 4.5.2. Given the 31.8% loss to follow‐up and the assumption of missing at random (MAR) supported by baseline comparisons, linear mixed‐effects model (LMM) with restricted maximum likelihood (REML) estimation was employed. This approach yields unbiased estimates under MAR by incorporating all available longitudinal data.

Missing baseline data were imputed using multiple imputation by chained equations (MICE). Imputation quality was assessed by examining convergence plots, comparing density distributions of observed and imputed values and inspecting box plots to ensure that the imputed distributions closely approximated the observed data.

After imputation, each of the five complete datasets was converted from wide to long format. To quantify the within‐individual correlation among repeated measures, a null model containing only a random intercept was first fitted and the adjusted ICC was computed. LMMs were then used to examine factors associated with QoL and its temporal trajectory. The models included a random intercept at the individual level and fixed effects for time (modelled using a natural cubic spline with 3 degrees of freedom). Baseline covariates were age, gender, education, smoking, alcohol drinking, marital status, employment, primary caregiver, family economic status, family history of stroke, prior stroke, NIHSS score and ADL score. Time‐varying covariates were exercise adherence score and mRS grade, which were measured at each follow‐up visit and modelled concurrently with QoL at the same visit.

Each model was fitted to the five imputed datasets separately and estimates were pooled using Rubin's rules. The overall significance of the time effect was assessed by combining likelihood ratio tests across imputed datasets. A sensitivity analysis using a delta‐adjusted pattern‐mixture model was conducted to confirm the robustness of the findings.

## 4 Results

### 4.1 Participant Characteristics

A total of 358 patients were initially recruited. After applying the exclusion criteria, 355 were enrolled at discharge (one patient was transferred to a rehabilitation hospital and two were discharged at the family's request). Figure 1 shows the numbers of patients who completed QoL assessments at each follow‐up time point and the reasons for loss to follow‐up. Overall, 242 patients (68.2%) completed the 24‐month follow‐up. All 355 patients contributed at least one QoL assessment, yielding a total of 1795 observations that were included in the LMM.

**FIGURE 1:**
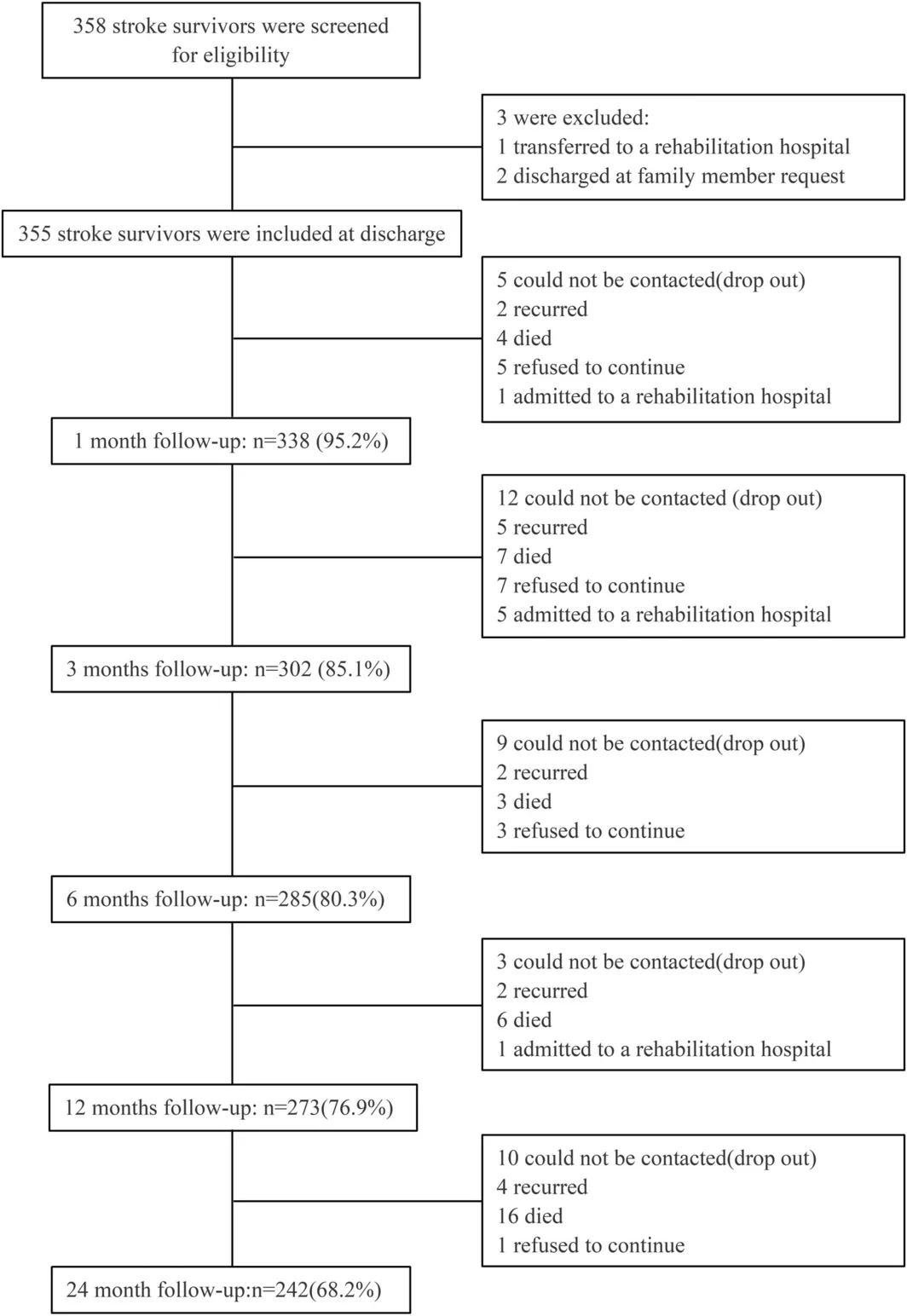
Flow‐chart of survivors' enrolment and follow‐up.

The mean age of the participants was 65.90 ± 11.92 years and 63.7% were male. The majority had a low educational level: 79.7% had not completed undergraduate education and 17.5% were illiterate. Baseline characteristics are summarized in Table 1.

**TABLE 1:** Baseline characteristics of participants (*N* = 355).

Characteristics	Value
Age, mean ± SD	65.90 ± 11.92
Gender, *n* (%)	
Male	226 (63.7)
Female	129 (36.3)
Education, *n* (%)	
Below bachelor's degree	283 (79.7)
Bachelor's degree	10 (2.8)
Illiterate	62 (17.5)
Smoking, *n* (%)	
Yes	174 (49.0)
No	181 (51.0)
Alcohol drinking, *n* (%)	
Yes	38 (10.7)
No	317 (89.3)
Marital status, *n* (%)	
Married	321 (90.4)
Unmarried	11 (3.1)
Divorced/Widowed	23 (6.5)
Employment, *n* (%)	
Retired	108 (30.4)
Employed	42 (11.8)
Unemployed	205 (57.7)
Primary caregiver, *n* (%)	
Spouse	142 (40.0)
Child	130 (36.6)
Nursing staff	83 (23.4)
Family economic status, *n* (%)	
Surplus	33 (9.3)
Balanced	281 (79.2)
Deficit	41 (11.5)
Family history of stroke, *n* (%)	
Yes	56 (15.8)
No	299 (84.2)
Prior stroke, *n* (%)	
Yes	72 (20.3)
No	283 (79.7)
NIHSS score, median (IQR)^a^	4.00 (2.00–6.00)
ADL score, mean ± SD	72.10 ± 30.58
Exercise adherence score at discharge, mean ± SD	41.95 ± 9.08
mRS grade at discharge, *n* (%)	
0 = No symptoms	15 (4.2)
1 = No significant disability	142 (40.0)
2 = Slight disability	43 (12.1)
3 = Moderate disability	34 (9.6)
4 = Moderately severe disability	93 (26.2)
5 = Severe disability	28 (7.9)
QOL score at discharge, mean ± SD	163.07 ± 32.96

*Note:* Continuous variables are expressed as mean ± standard deviation or median [interquartile range]; categorical variables as number (percentage).

Abbreviations: ADL, activities of daily living; mRS, modified Rankin Scale; NIHSS, National Institutes of Health Stroke Scale; QOL, quality of life.

^a^ The NIHSS score was missing in 58 patients (16.34%) at baseline; reported values are for the 297 patients with observed data. Multiple imputation was used in subsequent analyses.

When comparing baseline characteristics between completers (*n* = 242) and non‐completers (*n* = 113), significant differences were observed (Table S1). The findings indicate that attrition was systematically associated with greater disease severity and lower social support at baseline, supporting a MAR mechanism rather than missing completely at random (MCAR).

### 4.2 Missing Value Interpolation

Among the 355 participants, baseline NIHSS scores were missing for 58 cases (16.34%), while no missing data were observed for the remaining variables. After MICE imputation, the density curves of the observed and imputed NIHSS values across the five imputed datasets showed a high degree of overlap, with nearly identical medians and interquartile ranges, indicating satisfactory imputation quality (Figures S1, S2).

### 4.3 Factors Associated With QoL

The full model fixed‐effects results are presented in Table 2. After combining the results from the five imputed datasets following multiple imputation, factors significantly associated with QoL included: family history of stroke (Yes vs. No, *β* = −4.50, 95% CI: −7.62 to −1.37, *p* = 0.005), prior stroke (Yes vs. No, *β* = −3.16, 95% CI: −6.02 to −0.31, *p* = 0.030), NIHSS score (per 1‐point increase, *β* = −0.80, 95% CI: −1.22 to −0.38, *p* < 0.001), ADL score (per 1‐point increase, *β* = 0.17, 95% CI: 0.10 to 0.23, *p* < 0.001), exercise adherence score (per 1‐point increase, *β* = 0.82, 95% CI: 0.69 to 0.95, *p* < 0.001) and mRS grade with mRS 0 as the reference: mRS 1 (*β* = −3.71, 95% CI: −6.05 to −1.37, *p* = 0.002), mRS 2 (*β* = −15.12, 95% CI: −18.38 to −11.85, *p* < 0.001), mRS 3 (*β* = −22.00, 95% CI: −25.84 to −18.17, *p* < 0.001), mRS 4 (*β* = −43.13, 95% CI: −47.52 to −38.74, *p* < 0.001), mRS 5 (*β* = −70.71, 95% CI: −78.02 to −63.39, *p* < 0.001).

**TABLE 2:** Fixed effects from linear mixed‐effects model for QOL over 24 months (*N* = 355).

Variable	Β (Estimate)	SE	95% CI	*t v*alue	*p v*alue
(Intercept)**	152.14	7.85	136.72 to 167.55	19.37	< 0.001
Age (per year)	0.05	0.07	−0.08 to 0.19	0.77	0.439
Gender (Male vs. Female)	2.91	1.64	−0.31 to 6.13	1.77	0.077
Education (Ref: Below bachelor's degree)					
Bachelor's degree	1.61	3.54	−5.33 to 8.55	0.46	0.648
Illiterate	1.21	1.70	−2.11 to 4.54	0.72	0.474
Smoking (Yes vs. No)	−2.65	1.50	−5.59 to 0.30	−1.76	0.078
Alcohol drinking (Yes vs. No)	1.89	1.97	−1.97 to 5.76	0.96	0.336
Marital status (Ref: Married)					
Unmarried	1.65	3.44	−5.09 to 8.39	0.48	0.630
Divorced/Widowed	−3.53	2.81	−9.04 to 1.99	−1.25	0.210
Employment (Ref: Retired)					
Employed	1.85	2.48	−3.02 to 6.72	0.74	0.457
Unemployed	2.22	1.43	−0.58 to 5.02	1.55	0.120
Primary caregiver (Ref: Spouse)					
Child	−0.12	1.44	−2.94 to 2.71	−0.08	0.936
Nursing staff	−1.08	1.57	−4.17 to 2.00	−0.69	0.491
Family economic status (Ref: Surplus)					
Balanced	−3.52	2.05	−7.54 to 0.51	−1.71	0.087
Deficit	−2.67	2.64	−7.84 to 2.51	−1.01	0.312
Family history of stroke (Yes vs. No)*	−4.50	1.59	−7.62 to –1.37	−2.82	0.005
Prior stroke (Yes vs. No)*	−3.16	1.46	−6.02 to –0.31	−2.17	0.030
NIHSS score (per point)**	−0.80	0.21	−1.22 to –0.38	−3.84	< 0.001
ADL score (per point)**	0.17	0.03	0.10 to 0.23	5.05	< 0.001
Exercise adherence score (per point)**	0.82	0.07	0.69 to 0.95	12.34	< 0.001
mRS grade (Ref: 0)					
mRS1*	−3.71	1.19	−6.05 to –1.37	−3.10	0.002
mRS2**	−15.12	1.66	−18.38 to –11.85	−9.09	< 0.001
mRS3**	−22.00	1.95	−25.84 to –18.17	−11.26	< 0.001
mRS4**	−43.13	2.24	−47.52 to –38.74	−19.28	< 0.001
mRS5**	−70.71	3.73	−78.02 to –63.39	−18.95	< 0.001
Time trends					
ns (time_num, df = 3)1**	6.28	1.85	2.66 to 9.90	3.40	< 0.001
ns (time_num, df = 3)2**	73.41	1.99	69.51 to 77.31	36.90	< 0.001
ns (time_num, df = 3)3**	15.92	1.16	13.65 to 18.18	13.78	< 0.001

*Note:* The model included random intercepts for participants and was adjusted for time using natural cubic splines (df = 3); the overall time effect was significant (pooled likelihood ratio test: F(3, 741502.7) = 54.13, *p* < 0.001). Estimates were pooled from 5 multiple‐imputed datasets using Rubin's rules.

Abbreviations*: β*, unstandardized regression coefficient; ADL, activities of daily living; CI, confidence interval; mRS, modified Rankin Scale; NIHSS, National Institutes of Health Stroke Scale; QOL, quality of life; SE, standard error.

* *p* < 0.05.

** *p* < 0.001.

### 4.4 Trends in QOL Over Time

The natural cubic spline terms for time in the full model were all significant (Table 2) and the overall time effect was significant (combined likelihood ratio test: F(3, 741502.7) = 54.13, *p* < 0.001), confirming a nonlinear change in QoL over time after covariate adjustment. The plot of predicted marginal means revealed that QoL increased rapidly during the first 3 months after discharge, followed by a slower increase to its peak at 6 months (predicted mean 210.73, 95% CI: 206.35–215.13). However, QoL declined between 6 and 12 months and after 12 months, it showed a slow upward trend (Figure S3).

### 4.5 Sensitivity Analysis

We evaluated the robustness of the association between exercise adherence and QoL against nonrandom missingness using a delta‐adjusted pattern‐mixture model. In the primary analysis (Table 2), each 1‐point increase in exercise adherence was associated with a 0.82‐point increase in QoL (95% CI: 0.69–0.95, *p* < 0.001). In the delta‐adjusted sensitivity analysis, the corresponding estimate at Δ = 0 was 0.78 (95% CI: 0.64–0.92, *p* < 0.001). This sensitivity model additionally imputed missing QoL to permit delta adjustment, whereas the primary model used available observed QoL values (Table S2). Across a range of assumed QoL shifts for dropouts (Δ = ±10), the estimated effects remained consistently positive, with confidence intervals not crossing zero. When the assumed shift was set to ±5 points (within the minimal clinically important difference), the β estimates varied minimally (0.77–0.78), all with *p* < 0.001. The tipping point was not reached even at Δ = ±10, indicating that the association between exercise adherence and better long‐term QoL remained robust under substantial nonrandom missingness (Figure S4). These sensitivity analyses suggest that selective attrition is unlikely to materially alter the positive association between exercise adherence and QoL, thus supporting the reliability of our main findings.

## 5 Discussion

This study aimed to identify factors associated with QoL among stroke survivors within 2 years post‐discharge, with a particular focus on exercise adherence. In this observational cohort, the degree of disability and exercise adherence were independently associated with QoL during the two‐year follow‐up. In addition, family history of stroke and prior stroke were independently associated with poorer long‐term QoL.

The degree of disability was significantly associated with QoL among stroke survivors throughout the two‐year follow‐up. Higher NIHSS scores and mRS grades were significantly associated with lower QoL, whereas higher ADL scores were associated with better QoL. These findings suggest that the degree of physical disability is a major factor associated with QoL, consistent with existing evidence. Previous studies have shown that stroke severity (assessed by NIHSS and mRS) and ADL are independently associated with QoL among survivors at 3, 6 or 12 months post‐discharge (Fatema et al. 2022; Gräßel et al. 2019; Lee et al. 2022). For example, a cohort study of 2441 stroke survivors found that mRS and functional independence were independently associated with QoL at 6 months (Kim et al. 2023). In that study, ischemic stroke accounted for 83.94% of cases, the mean age was 62.13 ± 12.63 years and 62.43% of patients were male; these characteristics are consistent with those of the present study population. However, previous studies have mostly followed patients for less than 1 year, with few follow‐up time points (typically only one or two), limiting the ability to draw systematic and coherent inferences. Moreover, assessments of disability were largely restricted to baseline characteristics. In contrast, the present study extended follow‐up to 2 years, with repeated QoL assessments at 1, 3, 6, 12 and 24 months post‐discharge and found that both baseline functional impairment and the trajectory of functional disability over time were persistently and significantly associated with survivors' QoL. These findings address a key gap in the literature and further emphasise the necessity of long‐term interventions targeting functional recovery in rehabilitation nursing.

A family history of stroke and prior stroke were associated with poorer QoL within 2 years post‐discharge. A family history of stroke is a well‐established risk factor for recurrence (Zhang et al. 2022). Previous studies have indicated that it does not influence long‐term functional outcomes after rehabilitation (Błaż and Sarzyńska‐Długosz 2023). However, a family history not only reflects genetic susceptibility but may also indicate an adverse family lifestyle and environment. In this study, the observed long‐term association between family history and QoL may be explained by differences in rehabilitation behaviours or psychological status among survivors with a family history (Kim et al. 2025), which may in turn be related to overall QoL as measured by the SS‐QoL, a scale encompassing physical, psychological and social participation dimensions. Compared with first‐ever stroke survivors, those with prior stroke have preexisting irreversible neurological deficits and recovery following recurrent stroke may be slower with a more limited rehabilitation potential, thereby restricting participation in social life, including daily activities and interpersonal interactions (Lee et al. 2022) and negatively affecting long‐term QoL. Moreover, survivors with a family history of stroke or prior stroke often experience heightened concern and anxiety about stroke recurrence (Zhang et al. 2022). This psychological burden can lead to reduced social participation, decreased rehabilitation motivation, shortened sleep duration (Zhang et al. 2022) and other issues that hinder functional recovery and QoL improvement after stroke (Park and Yeom 2026). Therefore, these non‐modifiable risk factors should be considered in post‐discharge rehabilitation and care plans and combined exercise and psychological interventions should be initiated earlier for patients carrying these markers to maximise their long‐term QoL.

Functional exercise is a primary means of restoring ADL in stroke survivors and represents the preferred rehabilitation therapy after stroke. Adherence to functional rehabilitation exercises has been linked to better physical function and enhanced recovery, which contribute to a better QoL after stroke (Barbosa et al. 2022; Wang et al. 2024). Although most research has focused on the effects of different exercise modalities on post‐stroke outcomes (Dong et al. 2024; Fan et al. 2025; Jiang et al. 2025; Wei et al. 2024), recovery for survivors with disability is a long‐term process that requires sustained exercise. Exercise adherence—the extent to which patients consistently follow prescribed functional exercises—has received limited attention with respect to its association with QoL (Gelineau et al. 2022; Wang et al. 2024). The present findings extend the evidence in this field by showing that exercise adherence was significantly and independently associated with better QoL on average over the 24‐month follow‐up period in ischemic stroke survivors, suggesting that the degree of cooperation and adherence to functional exercise are significantly associated with their QoL at various stages of post‐stroke rehabilitation. Greater adherence correlates with improved QoL.

The mechanisms underlying the association between exercise adherence and long‐term QoL in stroke survivors may be explored through established nursing and behavioural theories. Based on Orem's self‐care theory (Hartweg and Metcalfe 2021), combined with self‐determination theory and habit formation theory, it becomes clear how nurses can effectively facilitate this transition process. According to Orem, nurses are responsible for assessing patients' self‐care deficits and providing appropriate care—wholly compensatory, partly compensatory or supportive‐educative—to help patients regain the ability to perform self‐care. During post‐stroke rehabilitation, the nursing approach shifts from wholly or partly compensatory care toward a supportive‐educative model. Nurses act as educators and motivators, helping patients acquire the knowledge and skills required for self‐care. At this stage, self‐determination theory and habit formation theory help explain the importance of sustained exercise adherence. From the perspective of self‐determination theory (Flannery 2017), sustained engagement in functional exercise can fulfil the basic psychological needs for autonomy and competence in stroke survivors. Satisfying these needs promotes the internalisation of motivation, shifting behavioural regulation from external sources to intrinsic motivation, thereby enhancing behavioural persistence, mental health and health outcomes (Ntoumanis and Moller 2025). Furthermore, the long‐term relationship between exercise adherence and QoL may be understood through habit formation theory. Habit formation theory (Gardner 2015) posits that repeated performance of functional exercises in a familiar home environment leads to increased automaticity, such that the behaviour is triggered by contextual cues (e.g., specific times or the presence of caregivers) with minimal deliberate effort or conscious intention. Once established, a habit is resistant to motivational fluctuations and environmental disruptions, a phenomenon referred to as automaticity (Verplanken and Orbell 2022). Understanding these mechanisms can inform the development of more effective long‐term care strategies.

From this integrated theoretical perspective, several recommendations for rehabilitation nursing practice guidelines can be proposed. First, guidelines should explicitly incorporate patient‐centered educational strategies (e.g., granting patients autonomy in selecting exercise modalities and enhancing their sense of competence through progressive goal setting and positive feedback) into standard rehabilitation nursing procedures. This theory‐informed supportive‐educative approach extends beyond mere information delivery and, more importantly, fosters patients' intrinsic motivation. Second, guidelines should emphasise that nursing interventions should focus not only on helping patients initially master exercise techniques but also on promoting behavioural automaticity over time, thereby facilitating the formation of exercise habits. This is essential for ensuring continuity of care: well‐designed transitional care plans, remote monitoring and community‐based reinforcement measures are all necessary to bridge the gap between hospital‐based rehabilitation and long‐term home care. Third, guidelines should highlight nurses' role in cultivating patients' self‐management capacity. Nurses are not merely exercise supervisors but facilitators of behaviour change, empowering patients to take ownership of their long‐term rehabilitation.

It is worth noting that the relationship between exercise adherence and QoL is likely mediated through both physical and psychological pathways. Physically, sustained adherence helps preserve or improve functional capacity and ADL, thereby reducing dependency and enhancing the physical dimension of QoL (Kwon et al. 2018). Psychologically, survivors with high exercise adherence may exhibit greater self‐efficacy (Yanfang et al. 2022), more positive coping styles (Jieqiong et al. 2019) and better adaptability to the rehabilitation process (Dharma et al. 2023), which may in turn facilitate rehabilitation outcomes (Wang et al. 2024) and lead to higher QoL after discharge (Bakhtiar et al. 2024; Slenders et al. 2024; Wang et al. 2024). Given that exercise adherence is a modifiable factor, targeted interventions to improve it could yield substantial benefits for stroke survivors.

Previous studies have indicated that exercise adherence in stroke survivors is significantly associated with personal attitudes/beliefs, social support, income level and access to modern information technologies (Mahmood et al. 2023; Sulwarajan et al. 2025). Therefore, during discharge education, nurses should focus on fostering a positive attitude toward exercise (e.g., highlighting the benefits of exercise or emphasizing the necessity and feasibility of exercise). Family members and friends should also be encouraged to participate in functional exercise instruction. Meanwhile, nurses can provide socioeconomically disadvantaged stroke survivors with greater access to exercise‐related information, such as free functional exercise videos and effective, low‐cost traditional Chinese exercises (e.g., Tai Chi, Baduanjin and Qigong). As nurses play a pivotal role in modern telerehabilitation (Wong et al. 2025), the use of mobile applications, wearable trackers and websites for remote post‐discharge exercise guidance represents a promising future direction (Sharififar et al. 2023; Sulwarajan et al. 2025). These measures may help nurses promote and sustain survivors' exercise adherence, which may in turn be associated with better QoL after discharge.

### 5.1 Implications for Nursing Practice

Based on the findings, the following implications for nursing practice are proposed:

1. *Integrate adherence assessment into long‐term follow‐up care*. Nurses should routinely assess exercise adherence during post‐discharge follow‐up, given its sustained association with stroke survivors' QoL.
2. *Foster intrinsic motivation through theory‐informed education*. Guidelines should adopt patient‐centered educational strategies that promote autonomy in exercise selection and enhance competence via progressive goal‐setting and positive feedback, thereby cultivating sustained motivation beyond simple instruction.
3. *Promote habit formation and ensure continuity of care*. Nurses should facilitate the transition from deliberate exercise to automatic behavioural routines through transitional care plans, remote monitoring and community‐based reinforcement, bridging hospital rehabilitation with long‐term home care.
4. *Leverage accessible resources and digital technologies*. Provide low‐cost exercise options (e.g., free instructional videos, Tai Chi, Ba Duan Jin, Qigong) for disadvantaged patients, while utilizing mobile apps, wearable trackers and telehealth platforms to support adherence and ultimately improve long‐term QoL.

### 5.2 Strength and Limitations

This study has several strengths and limitations. The main strength is the inclusion of a large cohort of 355 ischemic stroke survivors followed prospectively for 2 years (at discharge and at 1, 3, 6, 12 and 24 months post‐discharge). Another strength is the use of the SS‐QoL scale and a stroke‐specific exercise adherence scale (EAQ), which enabled more precise and comprehensive assessment of the primary outcomes. Additionally, LMM was employed to account for the correlation among repeated measures and to maximise the use of all available data, consequently limiting the impact of selective dropout. Furthermore, longitudinal follow‐up was conducted by two dedicated researchers and data were independently double‐entered and verified using EpiData software, effectively reducing data collection bias.

However, this study was conducted at a single hospital in Guangdong, China, which may limit the generalizability of the findings. First, the region's relatively high socioeconomic status and healthcare accessibility may have reduced financial barriers for patients, whereas those in resource‐limited settings may face greater economic constraints. Second, the strong familial support typical of the local cultural context differs from that in regions with weaker family cohesion, making direct replication uncertain. Future multicenter studies are warranted to confirm the broader applicability of these findings.

The original sample size justification was based on events‐per‐variable rules for logistic and Cox regression, which are not directly applicable to the linear mixed‐effects model used in the final analysis. A formal a priori power calculation for the linear mixed‐effects model was not performed and post hoc power calculations were not conducted because they are generally considered uninformative and are determined by the observed effect size. Nevertheless, the 95% confidence interval for the primary association between exercise adherence and QoL was relatively narrow and excluded the null value (*β* = 0.82, 95% CI: 0.69–0.95), indicating adequate precision for the estimated average longitudinal association. However, the possibility of insufficient power to detect smaller or time‐varying associations cannot be excluded.

In this study, exercise adherence was significantly associated with QoL. However, because the analytical model did not adjust for potential confounders such as depression, motivation and other psychological factors, the causal nature of the relationship between exercise adherence and QoL requires confirmation in future experimental studies, such as randomized controlled trials.

In addition to the potential selection bias introduced by the exclusion of patients who were discharged due to critical illness or who were transferred to a rehabilitation hospital at discharge, the 31.8% overall attrition rate over the two‐year follow‐up further compounded this limitation. Patients lost to follow‐up were disproportionately older and had more severe functional disability at baseline, indicating a non‐random pattern of dropout. Consequently, the inverse associations of advanced age and greater disease severity with long‐term QoL may have been underestimated and the observed association between exercise adherence and QoL may represent a conservative estimate of the true association in the general stroke population. Therefore, our findings are most generalizable to stroke survivors with mild‐to‐moderate disability and should be extrapolated with caution to very old adults (e.g., those aged ≥ 80 years) or to those with severe initial functional deficits. Future longitudinal studies employing targeted retention strategies and deliberate oversampling of severely affected patients are warranted to validate these relationships across the full spectrum of stroke severity.

Because no exercise adherence‐by‐time interaction was included in the primary model, the exercise adherence coefficient should therefore be interpreted as an average longitudinal association across the 24‐month follow‐up rather than a stage‐specific association. Future studies should incorporate exercise adherence‐by‐time interaction terms to examine potential time‐varying associations and to identify optimal timing for exercise‐based rehabilitation.

## 6 Conclusion

The results of this study indicate that family history of stroke, prior stroke, NIHSS score, ADL score, exercise adherence and mRS grade are independently associated with QoL in ischemic stroke survivors over the 2‐year post‐discharge period. Notably, exercise adherence showed a significant positive association with QoL on average over the 24‐month follow‐up period, addressing an important gap in the literature. These findings suggest that nurses should promote exercise adherence as a strategy to support post‐stroke QoL and rehabilitation nursing guidelines should emphasise the integration of exercise adherence into long‐term follow‐up, together with the timely implementation of targeted strategies (e.g., motivational support, engagement of family and friends, free and accessible exercise instruction and the use of digital health technologies) to sustain exercise adherence and thereby improve long‐term QoL.

## Author Contributions

**Jin Ziyuan:** conceptualization, methodology, software, data curation, investigation, validation, formal analysis, visualization, writing – original draft, writing – review and editing. **Zhang Hong:** supervision, funding acquisition, project administration, resources, writing – review and editing. **Liu Zhili:** writing – review and editing, resources, methodology, conceptualization. **Wu Yanchun:** writing – review and editing, resources, conceptualization. **Lin Hongchun:** methodology, writing – review and editing.

## Funding

The author(s) disclosed receipt of the following financial support for the research, authorship and/or publication of this article: This work was supported by the Guangdong Provincial Nurses' Association (grant number: gdshsxh2021b004).

## Ethics Statement

This study was approved by the Ethics Committee of the First Affiliated Hospital of Shantou University Medical College (approval number: SDFY‐EC‐SOP‐004‐3.0‐A06).

## Consent

Informed consent was signed by all study participants.

## Conflicts of Interest

The authors declare no conflicts of interest.

## Supporting information


**Data S1:** STROBE Statement—checklist of items that should be included in reports of observational studies.

**Figure S1:** Observed versus imputed NIHSS density distributions.
**Figure S2:** Comparison of statistics between observed and imputed NIHSS.
**Figure S3:** Predicted marginal means of stroke survivors' QoL over time.
**Figure S4:** Tipping point plot for the effect of exercise adherence on QoL.

**Table S1:** Comparison of baseline data between the completed group and the incomplete group.
**Table S2:** Comparison of primary and delta‐adjusted sensitivity analysis estimates for the association between exercise adherence and quality of life.

## Data Availability

The data that support the findings of this study are available from the corresponding author upon reasonable request.
