# Supplementary material for: Association Between Exercise Adherence and Long‐Term Quality of Life in Ischemic Stroke Survivors: A 2‐Year Prospective Cohort Study
Source: Nurs Open. 2026 Oct 30;13(11):e70965. doi: 10.1002/nop2.70965 (PMC13722777; doi:10.1002/nop2.70965)
Supplement: Supplementary file 1 [file NOP2-13-e70965-s001.docx]

Figure S1. Observed vs. imputed NIHSS density distributions

Figure S2. Comparison of statistics between observed and imputed NIHSS

Figure S3. Predicted marginal means of stroke survivors' QoL over time

Based on pooled estimates from multiple imputation with adjustment for all covariates;

Overall test of the time effect F (3, 741502.7)=54.13, P<0.001

Figure S4. Tipping point plot for the effect of exercise adherence on QoL.

Shaded areas represent 95% confidence intervals; red dashed line indicates the null effect (effect = 0).
