# Supplementary material for: Association Between Exercise Adherence and Long‐Term Quality of Life in Ischemic Stroke Survivors: A 2‐Year Prospective Cohort Study
Source: Nurs Open. 2026 Oct 30;13(11):e70965. doi: 10.1002/nop2.70965 (PMC13722777; doi:10.1002/nop2.70965)
Supplement: Supplementary file 2 [file NOP2-13-e70965-s002.docx]

Table 1. Comparison of baseline data between the completed group and the incomplete group

| Variable | Completed Group(n=242) | Incompleted Group(n=113) | Statistical Value | *p*-Value |
| --- | --- | --- | --- | --- |
| **Continuous variable** | **Mean±SD** | **Mean±SD** | **t** | **P** |
| Age^**^ | 64.39±11.10 | 69.13±11.33 | 3.548 | ＜0.001 |
| NIHSS score^**^ | 3.90±3.47 | 6.81±6.20 | 4.235 | ＜0.001 |
| ADL score^**^ | 77.64±26.60 | 60.22±34.98 | -4.698 | ＜0.001 |
| QOL score at discharge^**^ | 168.97±27.11 | 150.45±40.20 | -4.446 | ＜0.001 |
| Exercise adherence score at discharge^**^ | 43.37±7.63 | 38.90±11.04 | -3.889 | ＜0.001 |
| **Categorical Variable** | **n (%)** | **n (%)** | **χ²** | **P** |
| Gender |  |  | 2.799 | 0.094 |
| Male | 60.70 | 69.90 |  |  |
| Female | 39.30 | 30.10 |  |  |
| Education |  |  | 0.303 | 0.906 |
| Below bachelor's degree | 78.90 | 81.40 |  |  |
| Bachelor's degree | 2.90 | 2.70 |  |  |
| Illiterate | 18.20 | 15.90 |  |  |
| Smoking |  |  | 1.106 | 0.293 |
| Yes | 47.10 | 53.10 |  |  |
| No | 52.90 | 46.90 |  |  |
| Alcohol drinking |  |  | 0.163 | 0.686 |
| Yes | 11.20 | 9.70 |  |  |
| No | 88.80 | 90.30 |  |  |
| Marital status^**^ |  |  | 18.285 | ＜0.001 |
| Married | 94.20 | 82.30 |  |  |
| Unmarried | 3.30 | 2.70 |  |  |
| Divorced/Widowed | 2.50 | 15.00 |  |  |
| Employment |  |  | 5.813 | 0.055 |
| Retired | 28.10 | 35.40 |  |  |
| Employed | 14.50 | 6.20 |  |  |
| Unemployed | 57.40 | 58.40 |  |  |
| Primary caregiver^*^ |  |  | 7.952 | 0.019 |
| Spouse | 42.10 | 35.40 |  |  |
| Child | 31.80 | 46.90 |  |  |
| Nursing staff | 26.00 | 17.70 |  |  |
| Family economic status |  |  | 0.934 | 0.627 |
| Surplus | 8.70 | 10.60 |  |  |
| Balanced | 80.60 | 76.10 |  |  |
| Deficit | 10.70 | 13.30 |  |  |
| Family history of stroke |  |  | 0.326 | 0.568 |
| Yes | 83.50 | 85.80 |  |  |
| No | 16.50 | 14.20 |  |  |
| Prior stroke |  |  | 0.348 | 0.555 |
| Yes | 80.60 | 77.90 |  |  |
| No | 19.40 | 22.10 |  |  |
| mRS^**^ |  |  | 30.972 | ＜0.001 |
| No symptoms | 4.50 | 3.50 |  |  |
| No significant disability | 44.60 | 30.10 |  |  |
| Slight disability | 12.80 | 10.60 |  |  |
| Moderate disability | 8.70 | 11.50 |  |  |
| Moderately severe disability | 26.90 | 24.80 |  |  |
| Severe disability | 2.50 | 19.50 |  |  |

*Abbreviations:* NIHSS, National Institutes of Health Stroke Scale; ADL, activities of daily living; QOL, quality of life; mRS, modified Rankin Scale.

^*^P＜0.05，^**^P＜0.001.

Table 2. Comparison of primary and delta-adjusted sensitivity analysis estimates for the association between exercise adherence and quality of life.

| Model | Missing data handling | β for exercise adherence | 95% CI | *p*-Value |
| --- | --- | --- | --- | --- |
| Primary LMM | NIHSS MI; QOL available-case | 0.82 | 0.69-0.95 | <0.001 |
| Delta-adjusted PMM, Δ = 0 | NIHSS MI + QOL MI | 0.78 | 0.64-0.92 | <0.001 |
| Δ = ±5 | NIHSS MI + QOL MI + delta | 0.77-0.78 | 95% CIs did not cross zero | <0.001 |
| Δ = ±10 | NIHSS MI + QOL MI + delta | 0.76-0.78 | 95% CIs did not cross zero | <0.001 |

Note: The primary model imputed only missing NIHSS, whereas QoL was analyzed using available observed values. Because delta adjustment requires a complete QoL outcome, the sensitivity model additionally multiply imputed missing QoL; therefore, the Δ = 0 estimate is not expected to be identical to the primary estimate.
