# Supplementary material for: Association Between Exercise Adherence and Long‐Term Quality of Life in Ischemic Stroke Survivors: A 2‐Year Prospective Cohort Study
Source: Nurs Open. 2026 Oct 30;13(11):e70965. doi: 10.1002/nop2.70965 (PMC13722777; doi:10.1002/nop2.70965)
Supplement: Supplementary file 3 [file NOP2-13-e70965-s003.docx]

STROBE Statement—checklist of items that should be included in reports of observational studies

|  | Item No. | Recommendation | Page  No. | Relevant text from manuscript |
| --- | --- | --- | --- | --- |
| **Title and abstract** | 1 | (*a*) Indicate the study’s design with a commonly used term in the title or the abstract | 1 (Line 1-2; Line 6) | A 2-Year Prospective Cohort Study; a longitudinal prospective study |
|  |  | (*b*) Provide in the abstract an informative and balanced summary of what was done and what was found | 1 (Line 8-10; Line 14-16) | Methods: Participants were enrolled during hospitalization and followed up at discharge and at 1, 3, 6, 12, and 24 months post-discharge via questionnaires. Linear mixed-effects models were used to identify factors associated with quality of life.  Results: Of 355 enrolled stroke survivors, 242 (68.2%) completed the two-year follow-up. Functional disability, exercise adherence, family history of stroke, and prior stroke were independently associated with long-term quality of life. |
| Introduction | | | |  |
| Background/rationale | 2 | Explain the scientific background and rationale for the investigation being reported | 4 (Line 13-25)-5 (Line 1-18) | Numerous factors affecting QoL in stroke survivors have been identified, such as age (Segerdahl et al., 2023; Youkee et al., 2024), marital status (Ayasrah et al., 2024; Kim et al., 2023), education (Wijesiri et al., 2023), comorbidity (Diegoli et al., 2023; Rosa et al., 2023), functional independence (Braga et al., 2024), socioeconomic status (Sun et al., 2023). Among these, the degree of physical disability has been consistently associated with QoL (Barbosa et al., 2022; Braga et al., 2024; Diegoli et al., 2023; Gurková et al., 2023; Kim et al., 2023). This association may be partly explained by its relationship with self-efficacy (Dharma et al., 2023), coping style (van Mierlo et al., 2018) and exercise adherence.  Exercise adherence is defined as “a person’s adherence with a prescribed or self-prescribed fitness program” (Gelineau et al., 2022). Existing evidence indicates that exercise adherence in stroke survivors is shaped by a multidimensional network of determinants spanning psychological, social, and environmental domains. Psychological factors including subjective norms, attitudes, and perceived behavioral control predict post-stroke exercise intention, with self-efficacy representing the most consistent and direct predictor of exercise adherence in community-dwelling stroke survivors (Lin et al., 2022; Zhou et al., 2023). Rehabilitation expectations and subjective well-being may also be positively associated with adherence, whereas depressive symptoms and physical impairments are negatively associated with it (Liu et al., 2025; Xing et al., 2025; Zhang et al., 2023). In addition, family-related factors—particularly caregiver capacity and emotional support—play a crucial role in facilitating home-based exercise adherence among older stroke patients (Zhang et al., 2023). At the rehabilitation environment level, individually tailored exercise prescriptions, ongoing monitoring and feedback mechanisms, and supportive organizational policies have been identified as key enablers of sustained adherence (Liu et al., 2025; Vadas et al., 2022; Zhang et al., 2023). Given the pivotal role of exercise adherence in post-stroke recovery, a growing body of research has sought to identify its determinants. However, few studies have directly examined the prospective association between exercise adherence and long-term QoL. To address this research gap, we aimed to explore factors, especially exercise adherence, that are associated with QoL in stroke survivors at discharge and 1, 3, 6, 12, and 24 months post-discharge. We hypothesized that exercise adherence would be independently associated with long-term QoL among stroke survivors, after adjustment for demographic factors and functional disability. |
| Objectives | 3 | State specific objectives, including any prespecified hypotheses | 5 (Line 14-18) | To address this research gap, we aimed to explore factors, especially exercise adherence, that are associated with QoL in stroke survivors at discharge and 1, 3, 6, 12, and 24 months post-discharge. We hypothesized that exercise adherence would be independently associated with long-term QoL among stroke survivors, after adjustment for demographic factors and functional disability. |
| Methods | | | |  |
| Study design | 4 | Present key elements of study design early in the paper | 5 (Line 21) | This prospective observational study was conducted at REDACTED, Guangdong, China. |
| Setting | 5 | Describe the setting, locations, and relevant dates, including periods of recruitment, exposure, follow-up, and data collection | 5-8 | Participants and setting; Sample size and precision; Measurements; Data collection |
| Participants | 6 | (*a*) *Cohort study*—Give the eligibility criteria, and the sources and methods of selection of participants. Describe methods of follow-up  *Case-control study*—Give the eligibility criteria, and the sources and methods of case ascertainment and control selection. Give the rationale for the choice of cases and controls  *Cross-sectional study*—Give the eligibility criteria, and the sources and methods of selection of participants | 5-6; 8 (Line 13-24) | Inclusion criteria were as follows: (a) diagnosis of ischemic stroke confirmed by computed tomography or magnetic resonance imaging of the head, according to the diagnostic criteria of the Guidelines for the Prevention and Treatment of Cerebrovascular Diseases in China (Mingli, 2006); (b) age ≥18 years; (c) ability to cooperate in completing the questionnaire at discharge and during the follow-up period; (d) possession of a mobile phone to enable completion of follow-up; and (e) voluntary participation in the study. Exclusion criteria were as follows: (a) discharge due to critical illness or at the family’s request, and (b) transfer to a rehabilitation hospital at discharge.  Withdrawal criteria were as follows: (a) inability to contact the participant after two phone calls per day for three consecutive days；(b) stroke recurrence or death; (c) refusal to continue after the first follow-up; (d) admission to a rehabilitation hospital after discharge; or (e) hospitalization for other serious illnesses after the first follow-up.  Data collection  Two trained data collectors gathered all study data from participants. Follow-up data on exercise adherence, QoL, and mRS grade were collected at 1, 3, 6, 12, and 24 months post-discharge.  To ensure data quality, the following measures were implemented: (a) both data collectors underwent standardized training and used consistent wording and explanations during questionnaire administration; (b) to enhance follow-up adherence and minimize loss to follow-up, participants were reinformed about the follow-up procedures and study duration after completing the baseline questionnaire, and their contact information was reconfirmed; (c) the same data collection method was used at each time point to reduce bias arising from procedural variations; (d) all data were independently double-entered and cross-checked by two researchers using EpiData version 3.1 to minimize data entry errors. |
|  |  | (*b*) *Cohort study*—For matched studies, give matching criteria and number of exposed and unexposed  *Case-control study*—For matched studies, give matching criteria and the number of controls per case | Not applicable | This study is not a matched study. |
| Variables | 7 | Clearly define all outcomes, exposures, predictors, potential confounders, and effect modifiers. Give diagnostic criteria, if applicable | 6 (Line 24-25)-7 (Line 1-5) | Sociodemographic data of participants, including age, gender, education, smoking, alcohol drinking, marital status, employment, primary caregiver, family economic status, and family history of stroke, were collected using structured questionnaires. Clinical data, including prior stroke, National Institute of Health Stroke Scale (NIHSS) score, were extracted from medical records. Additional variables, including Activities of Daily Living (ADL) score, exercise adherence, modified Rankin Scale (mRS) grade, and QoL were assessed using the scales described below. |
| Data sources/ measurement | 8* | For each variable of interest, give sources of data and details of methods of assessment (measurement). Describe comparability of assessment methods if there is more than one group | *7-8* | *Barthel index scale; Exercise Adherence Questionnaire; Stroke-specific Quality of Life Scale; Modified Rankin Scale* |
| Bias | 9 | Describe any efforts to address potential sources of bias | 8-9 | To ensure data quality, the following measures were implemented: (a) both data collectors underwent standardized training and used consistent wording and explanations during questionnaire administration; (b) to enhance follow-up adherence and minimize loss to follow-up, participants were reinformed about the follow-up procedures and study duration after completing the baseline questionnaire, and their contact information was reconfirmed; (c) the same data collection method was used at each time point to reduce bias arising from procedural variations; (d) all data were independently double-entered and cross-checked by two researchers using EpiData version 3.1 to minimize data entry errors. |
| Study size | 10 | Explain how the study size was arrived at | 6 (Line 12-22) | Sample size and precision  The present study used data from an existing longitudinal cohort of ischemic stroke survivors. Accordingly, the sample size was determined by the number of eligible participants with available follow-up data rather than by a formal a priori sample size calculation for a linear mixed-effects model. Given that power calculations for linear mixed-effects models incorporating repeated measures and natural spline terms are statistically complex and highly dependent on assumptions regarding variance components and within-person correlation, we prioritized the precision of the estimated associations, as reflected by 95% confidence intervals, over conventional power thresholds. The primary association between exercise adherence and QoL was estimated with reasonable precision (β= 0.82, 95% CI: 0.69-0.95), and the confidence interval excluded the null value. |

Continued on next page

| Quantitative variables | 11 | Explain how quantitative variables were handled in the analyses. If applicable, describe which groupings were chosen and why | 9 (Line 10-19) | After imputation, each of the five complete datasets was converted from wide to long format. To quantify the within-individual correlation among repeated measures, a null model containing only a random intercept was first fitted, and the adjusted ICC was computed. LMMs were then used to examine factors associated with QoL and its temporal trajectory. The models included a random intercept at the individual level and fixed effects for time (modeled using a natural cubic spline with 3 degrees of freedom). Baseline covariates were age, gender, education, smoking, alcohol drinking, marital status, employment, primary caregiver, family economic status, family history of stroke, prior stroke, National Institute of Health stroke scale (NIHSS) score, and ADL score. Time-varying covariates were exercise adherence score and mRS grade, which were measured at each follow-up visit and modeled concurrently with QoL at the same visit. |
| --- | --- | --- | --- | --- |
| Statistical methods | 12 | (*a*) Describe all statistical methods, including those used to control for confounding | 9 (Line 1-23) | Statistical analyses were performed using R version 4.5.2. Given the 31.8% loss to follow-up and the assumption of missing at random (MAR) supported by baseline comparisons, Linear mixed-effects model (LMM) with restricted maximum likelihood (REML) estimation was employed. This approach yields unbiased estimates under MAR by incorporating all available longitudinal data.  Missing baseline data were imputed using multiple imputation by chained equations (MICE). Imputation quality was assessed by examining convergence plots, comparing density distributions of observed and imputed values, and inspecting box plots to ensure that the imputed distributions closely approximated the observed data.  After imputation, each of the five complete datasets was converted from wide to long format. To quantify the within-individual correlation among repeated measures, a null model containing only a random intercept was first fitted, and the adjusted ICC was computed. LMMs were then used to examine factors associated with QoL and its temporal trajectory. The models included a random intercept at the individual level and fixed effects for time (modeled using a natural cubic spline with 3 degrees of freedom). Baseline covariates were age, gender, education, smoking, alcohol drinking, marital status, employment, primary caregiver, family economic status, family history of stroke, prior stroke, National Institute of Health stroke scale (NIHSS) score, and ADL score. Time-varying covariates were exercise adherence score and mRS grade, which were measured at each follow-up visit and modeled concurrently with QoL at the same visit.  Each model was fitted to the five imputed datasets separately, and estimates were pooled using Rubin's rules. The overall significance of the time effect was assessed by combining likelihood ratio tests across imputed datasets. A sensitivity analysis using a delta‑adjusted pattern‑mixture model was conducted to confirm the robustness of the findings. |
|  |  | (*b*) Describe any methods used to examine subgroups and interactions | Not applicable | This study did not analyse subgroups or interactions. |
|  |  | (*c*) Explain how missing data were addressed | 9 (Line 6-9) | Missing baseline data were imputed using multiple imputation by chained equations (MICE). Imputation quality was assessed by examining convergence plots, comparing density distributions of observed and imputed values, and inspecting box plots to ensure that the imputed distributions closely approximated the observed data. |
|  |  | (*d*) *Cohort study*—If applicable, explain how loss to follow-up was addressed  *Case-control study*—If applicable, explain how matching of cases and controls was addressed  *Cross-sectional study*—If applicable, describe analytical methods taking account of sampling strategy | 9 (Line 1-5) | Given the 31.8% loss to follow-up and the assumption of missing at random (MAR) supported by baseline comparisons, Linear mixed-effects model (LMM) with restricted maximum likelihood (REML) estimation was employed. This approach yields unbiased estimates under MAR by incorporating all available longitudinal data. |
|  |  | (*e*) Describe any sensitivity analyses | 11-12 | We evaluated the robustness of the association between exercise adherence and QoL against nonrandom missingness using a delta-adjusted pattern-mixture model. In the primary analysis (Table 2), each 1-point increase in exercise adherence was associated with a 0.82-point increase in QoL (95% CI: 0.69-0.95, P < 0.001). In the delta-adjusted sensitivity analysis, the corresponding estimate at Δ=0 was 0.78 (95% CI: 0.64–0.92, P < 0.001). This sensitivity model additionally imputed missing QoL to permit delta adjustment, whereas the primary model used available observed QoL values (Supplementary Material, Table 2). Across a range of assumed QoL shifts for dropouts (Δ = ±10), the estimated effects remained consistently positive, with confidence intervals not crossing zero. When the assumed shift was set to ±5 points (within the minimal clinically important difference), the β estimates varied minimally (0.77-0.78), all with P < 0.001. The tipping point was not reached even at Δ = ±10, indicating that the association between exercise adherence and better long-term QoL remained robust under substantial nonrandom missingness (Supplementary Material, Figure 4). These sensitivity analyses suggest that selective attrition is unlikely to materially alter the positive association between functional exercise adherence and QoL, thus supporting the reliability of our main findings. |
| Results | | | | |
| Participants | 13* | (a) Report numbers of individuals at each stage of study—eg numbers potentially eligible, examined for eligibility, confirmed eligible, included in the study, completing follow-up, and analysed | 10 (Line 1-7); Figure 1. | A total of 358 patients were initially recruited. After applying the exclusion criteria, 355 were enrolled at discharge (one patient was transferred to a rehabilitation hospital and two were discharged at the family's request). Figure 1 shows the numbers of patients who completed QoL assessments at each follow-up time point and the reasons for loss to follow-up. Overall, 242 patients (68.2%) completed the 24-month follow-up. All 355 patients contributed at least one QoL assessment, yielding a total of 1,795 observations that were included in the LMM. |
|  |  | (b) Give reasons for non-participation at each stage | Figure 1 | none |
|  |  | (c) Consider use of a flow diagram | Figure 1 | none |
| Descriptive data | 14* | (a) Give characteristics of study participants (eg demographic, clinical, social) and information on exposures and potential confounders | 10 (Line 8-10); Table 1. | The mean age of the participants was 65.90 ± 11.92 years, and 63.7% were male. The majority had a low educational level: 79.7% had not completed undergraduate education, and 17.5% were illiterate. Baseline characteristics are summarized in Table 1. |
|  |  | (b) Indicate number of participants with missing data for each variable of interest | 10 (Line 17-18) | Among the 355 participants, baseline NIHSS scores were missing for 58 cases (16.34%), while no missing data were observed for the remaining variables. |
|  |  | (c) *Cohort study*—Summarise follow-up time (eg, average and total amount) | 10 (Line 5), Figure 1 | Overall, 242 patients (68.2%) completed the 24-month follow-up. |
| Outcome data | 15* | *Cohort study*—Report numbers of outcome events or summary measures over time | Figure 1 | none |
|  |  | *Case-control study—*Report numbers in each exposure category, or summary measures of exposure | Not applicable | This study is not a case-control study. |
|  |  | *Cross-sectional study—*Report numbers of outcome events or summary measures | Not applicable | This study is not a case-control study. |
| Main results | 16 | (*a*) Give unadjusted estimates and, if applicable, confounder-adjusted estimates and their precision (eg, 95% confidence interval). Make clear which confounders were adjusted for and why they were included | 10 (Line 23-25)-11 (Line 1-8), Table 2. | The full model fixed-effects results are presented in Table 2. After combining the results from the five imputed datasets following multiple imputation, factors significantly associated with QoL included: family history of stroke (Yes vs. No, β = -4.50, 95%, CI: -7.62 to -1.37, P = 0.005), prior stroke (Yes vs. No, β = -3.16, 95% CI: -6.02 to -0.31, P = 0.030), NIHSS score (per 1-point increase, β = -0.80, 95% CI: -1.22 to -0.38, P < 0.001), ADL score (per 1-point increase, β = 0.17, 95% CI: 0.10 to 0.23, P < 0.001), exercise adherence score (per 1-point increase, β = 0.82, 95% CI: 0.69 to 0.95, P < 0.001), and mRS grade with mRS 0 as the reference: mRS 1 (β = -3.71, 95% CI: -6.05 to -1.37, P = 0.002), mRS 2 (β = -15.12, 95% CI: -18.38 to -11.85, P < 0.001), mRS 3 (β = -22.00, 95% CI: -25.84 to -18.17, P < 0.001), mRS 4 (β = -43.13, 95% CI: -47.52 to -38.74, P < 0.001), mRS 5 (β = -70.71, 95% CI: -78.02 to -63.39, P < 0.001). |
|  |  | (*b*) Report category boundaries when continuous variables were categorized | Not applicable | Continuous variables were not categorised in this study |
|  |  | (*c*) If relevant, consider translating estimates of relative risk into absolute risk for a meaningful time period | Not applicable | This study did not estimate relative risks (RR, OR or HR) for any binary outcomes. |

Continued on next page

| Other analyses | 17 | Report other analyses done—eg analyses of subgroups and interactions, and sensitivity analyses | 11 (Line 17-25)-12 (Line 1-8) | Sensitivity analysis  We evaluated the robustness of the association between exercise adherence and QoL against nonrandom missingness using a delta-adjusted pattern-mixture model. In the primary analysis (Table 2), each 1-point increase in exercise adherence was associated with a 0.82-point increase in QoL (95% CI: 0.69-0.95, P < 0.001). In the delta-adjusted sensitivity analysis, the corresponding estimate at Δ=0 was 0.78 (95% CI: 0.64–0.92, P < 0.001). This sensitivity model additionally imputed missing QoL to permit delta adjustment, whereas the primary model used available observed QoL values (Supplementary Material, Table 2). Across a range of assumed QoL shifts for dropouts (Δ = ±10), the estimated effects remained consistently positive, with confidence intervals not crossing zero. When the assumed shift was set to ±5 points (within the minimal clinically important difference), the β estimates varied minimally (0.77-0.78), all with P < 0.001. The tipping point was not reached even at Δ = ±10, indicating that the association between exercise adherence and better long-term QoL remained robust under substantial nonrandom missingness (Supplementary Material, Figure 4). These sensitivity analyses suggest that selective attrition is unlikely to materially alter the positive association between functional exercise adherence and QoL, thus supporting the reliability of our main findings. |
| --- | --- | --- | --- | --- |
| Discussion | | | | |
| Key results | 18 | Summarise key results with reference to study objectives | 12 (Line 10-14) | This study aimed to identify factors associated with QoL among stroke survivors within two years post-discharge, with a particular focus on exercise adherence. In this observational cohort, the degree of disability and exercise adherence were independently associated with QoL during the two-year follow-up. In addition, family history of stroke and prior stroke were independently associated with poorer long-term QoL. |
| Limitations | 19 | Discuss limitations of the study, taking into account sources of potential bias or imprecision. Discuss both direction and magnitude of any potential bias | 18-20 | However, this study was conducted at a single hospital in Guangdong, China, which may limit the generalizability of the findings. First, the region’s relatively high socioeconomic status and healthcare accessibility may have reduced financial barriers for patients, whereas those in resource-limited settings may face greater economic constraints. Second, the strong familial support typical of the local cultural context differs from that in regions with weaker family cohesion, making direct replication uncertain. Future multicenter studies are warranted to confirm the broader applicability of these findings.  The original sample size justification was based on events-per-variable rules for logistic and Cox regression, which are not directly applicable to the linear mixed-effects model used in the final analysis. A formal a priori power calculation for the linear mixed-effects model was not performed, and post-hoc power calculations were not conducted because they are generally considered uninformative and are determined by the observed effect size. Nevertheless, the 95% confidence interval for the primary association between exercise adherence and QoL was relatively narrow and excluded the null value (β = 0.82, 95% CI: 0.69-0.95), indicating adequate precision for the estimated average longitudinal association. However, the possibility of insufficient power to detect smaller or time-varying associations cannot be excluded.  In this study, exercise adherence was significantly associated with QoL. However, because the analytical model did not adjust for potential confounders such as depression, motivation, and other psychological factors, the causal nature of the relationship between exercise adherence and QoL requires confirmation in future experimental studies, such as randomized controlled trials.  In addition to the potential selection bias introduced by the exclusion of patients who were discharged due to critical illness or who were transferred to a rehabilitation hospital at discharge, the 31.8% overall attrition rate over the two-year follow-up further compounded this limitation. Patients lost to follow-up were disproportionately older and had more severe functional disability at baseline, indicating a non-random pattern of dropout. Consequently, the inverse associations of advanced age and greater disease severity with long-term QoL may have been underestimated, and the observed association between exercise adherence and QoL may represent a conservative estimate of the true association in the general stroke population. Therefore, our findings are most generalizable to stroke survivors with mild-to-moderate disability and should be extrapolated with caution to very old adults (e.g., those aged ≥80 years) or to those with severe initial functional deficits. Future longitudinal studies employing targeted retention strategies and deliberate oversampling of severely affected patients are warranted to validate these relationships across the full spectrum of stroke severity.  Because no exercise adherence-by-time interaction was included in the primary model, the exercise adherence coefficient should therefore be interpreted as an average longitudinal association across the 24-month follow-up rather than a stage-specific association. Future studies should incorporate exercise adherence-by-time interaction terms to examine potential time-varying associations and to identify optimal timing for exercise-based rehabilitation. |
| Interpretation | 20 | Give a cautious overall interpretation of results considering objectives, limitations, multiplicity of analyses, results from similar studies, and other relevant evidence | 20 (Line 7-17) | The results of this study indicate that family history of stroke, prior stroke, NIHSS score, ADL score, exercise adherence, and mRS grade are independently associated with QoL in ischemic stroke survivors over the 2-year post-discharge period. Notably, exercise adherence showed a significant positive association with QoL on average over the 24-month follow-up period, addressing an important gap in the literature. These findings suggest that nurses should promote exercise adherence as a strategy to support post-stroke QoL, and rehabilitation nursing guidelines should emphasize the integration of exercise adherence into long-term follow-up, together with the timely implementation of targeted strategies (e.g., motivational support, engagement of family and friends, free and accessible exercise instruction, and the use of digital health technologies) to sustain exercise adherence and thereby improve long-term QoL. |
| Generalisability | 21 | Discuss the generalisability (external validity) of the study results | 19 (Line 20-22) | Therefore, our findings are most generalizable to stroke survivors with mild-to-moderate disability and should be extrapolated with caution to very old adults (e.g., those aged ≥80 years) or to those with severe initial functional deficits. |
| Other information | |  | | |
| Funding | 22 | Give the source of funding and the role of the funders for the present study and, if applicable, for the original study on which the present article is based | Title page | The author(s) disclosed receipt of the following financial support for the research, authorship, and/or publication of this article: This work was supported by the REDACTED [grant number REDACTED]. |

*Give information separately for cases and controls in case-control studies and, if applicable, for exposed and unexposed groups in cohort and cross-sectional studies.

**Note:** An Explanation and Elaboration article discusses each checklist item and gives methodological background and published examples of transparent reporting. The STROBE checklist is best used in conjunction with this article (freely available on the Web sites of PLoS Medicine at http://www.plosmedicine.org/, Annals of Internal Medicine at http://www.annals.org/, and Epidemiology at http://www.epidem.com/). Information on the STROBE Initiative is available at www.strobe-statement.org.
